# Phase partitioning of the neutrophil oxidative burst is coordinated by accessory pathways of glucose metabolism and mitochondrial activity

**DOI:** 10.1016/j.jbc.2024.108091

**Published:** 2024-12-13

**Authors:** Tyler Jobe, Jonah Stephan, Collin K. Wells, Maleesha De Silva, Pawel K. Lorkiewicz, Bradford G. Hill, Marcin Wysoczynski

**Affiliations:** 1 Center for Cardiometabolic Science, Christina Lee Brown Envirome Institute, University of Louisville, Louisville, Kentucky; 2 Department of Physiology, School of Medicine, University of Louisville, Louisville, Kentucky; 3 Department of Biochemistry, School of Medicine, University of Louisville, Louisville, Kentucky

**Keywords:** innate immunity, glycolysis, mitochondria, reactive oxygen species, pathogen

## Abstract

Neutrophils are a part of the innate immune system and produce reactive oxygen species (ROS) to extinguish pathogens. The major source of ROS in neutrophils is NADPH oxidase, which is fueled by NADPH generated *via* the pentose phosphate pathway; however, it is unclear how other accessory glucose metabolism pathways and mitochondrial activity influence the respiratory burst. We examined the temporal dynamics of the respiratory burst and delineated how metabolism changes over time after neutrophil activation. Bone marrow–derived neutrophils were stimulated with phorbol 12-myristate 13-acetate, and the respiratory burst was measured *via* extracellular flux analysis. Metabolomics experiments utilizing ^13^C_6_-glucose highlighted the activation of glycolysis as well as ancillary pathways of glucose metabolism in activated neutrophils. Phorbol 12-myristate 13-acetate stimulation acutely increased ^13^C enrichment into glycerol 3-phosphate (G3P) and citrate, whereas increases in ^13^C enrichment in the glycogen intermediate, UDP-hexose, and end products of the hexosamine and serine biosynthetic pathways occurred only during the late phase of the oxidative burst. Targeted inhibition of the G3P shuttle, glycogenolysis, serine biosynthesis, and mitochondrial respiration demonstrated that the G3P shuttle contributes to the general magnitude of ROS production; that glycogen contributes solely to the early respiratory burst; and that the serine biosynthetic pathway activity and complex III-driven mitochondrial activity influence respiratory burst duration. Collectively, these results show that the neutrophil oxidative burst is highly dynamic, with coordinated changes in metabolism that control the initiation, magnitude, and duration of ROS production.

Neutrophils are short-lived and terminally differentiated immune cells that play a crucial role in innate immunity (1, 2). Upon sensing signals associated with infection or injury, neutrophils extravasate into tissues and rapidly activate a series of effector functions to eliminate pathogens. Activated neutrophils produce copious amounts of reactive oxygen species (ROS), which not only contribute directly to bacterial killing but also activate granular proteases, induce neutrophil extracellular trap release, and influence the production of proinflammatory cytokines (3, 4). Because of their terminally differentiated state and short lifespan, neutrophils have a limited ability to mount transcriptional responses in response to pathogenic signals; however, they have an extraordinary capacity to allocate glucose-derived carbon to the oxidative pentose phosphate pathway (oxPPP), which regenerates NADPH and fuels the NADPH oxidase (Nox)–mediated respiratory burst. Given the importance of a rapid response to pathogens, it is likely that neutrophils leverage other forms of metabolic control to titrate the nature and extent of their response to infection or injury.

Compared with other immune cells, neutrophils have a unique metabolic fingerprint, especially in response to pathogenic stimuli (5, 6, 7). Previous studies show that neutrophils facilitate use of a pentose cycle, in which downstream PPP metabolites re-enter glycolysis at the levels of glyceraldehyde 3-phosphate and fructose 6-phosphate (F6P). *Via* reversal of glucose phosphate isomerase flux (8), the F6P is then converted to glucose 6-phosphate (G6P), which then re-enters the oxPPP to maximize NADPH regeneration, leaving glyceraldehyde 3-phosphate to be metabolized in the payoff phase of glycolysis. Furthermore, recent studies show that activated neutrophils exploit glycogen stores to regulate migratory activity and bacterial killing (9, 10), with gluconeogenesis used to support glycogen synthesis. Neutrophils may also leverage other metabolic pathways to control their effector functions. For example, the amphibolic nature of several 6-carbon and 3-carbon metabolic intermediates ([*e.g.*, G6P, F6P, dihydroxyacetone phosphate [DHAP], and 3-phosphoglycerate [3PG]) provide the ability to finely control glucose carbon fate, which could be critical to regulation of fundamental facets of neutrophil function such as the respiratory burst.

In this study, we used extracellular flux analysis and stable isotope metabolomics to understand the coordinated metabolic changes that occur with the respiratory burst. Using targeted pharmacological inhibition, we also assessed the impact of accessory pathways of glucose catabolism and mitochondrial activity on the duration and amplitude of neutrophil ROS production. Our findings indicate that glycogen catabolism, the glycerol-3 phosphate shuttle, serine biosynthesis, and mitochondrial activity shape the initiation, magnitude, and duration of the respiratory burst in activated neutrophils, highlighting unique metabolic points of control over neutrophil function.

## Results

### Neutrophil oxidative burst dynamics

**Figure 1:**
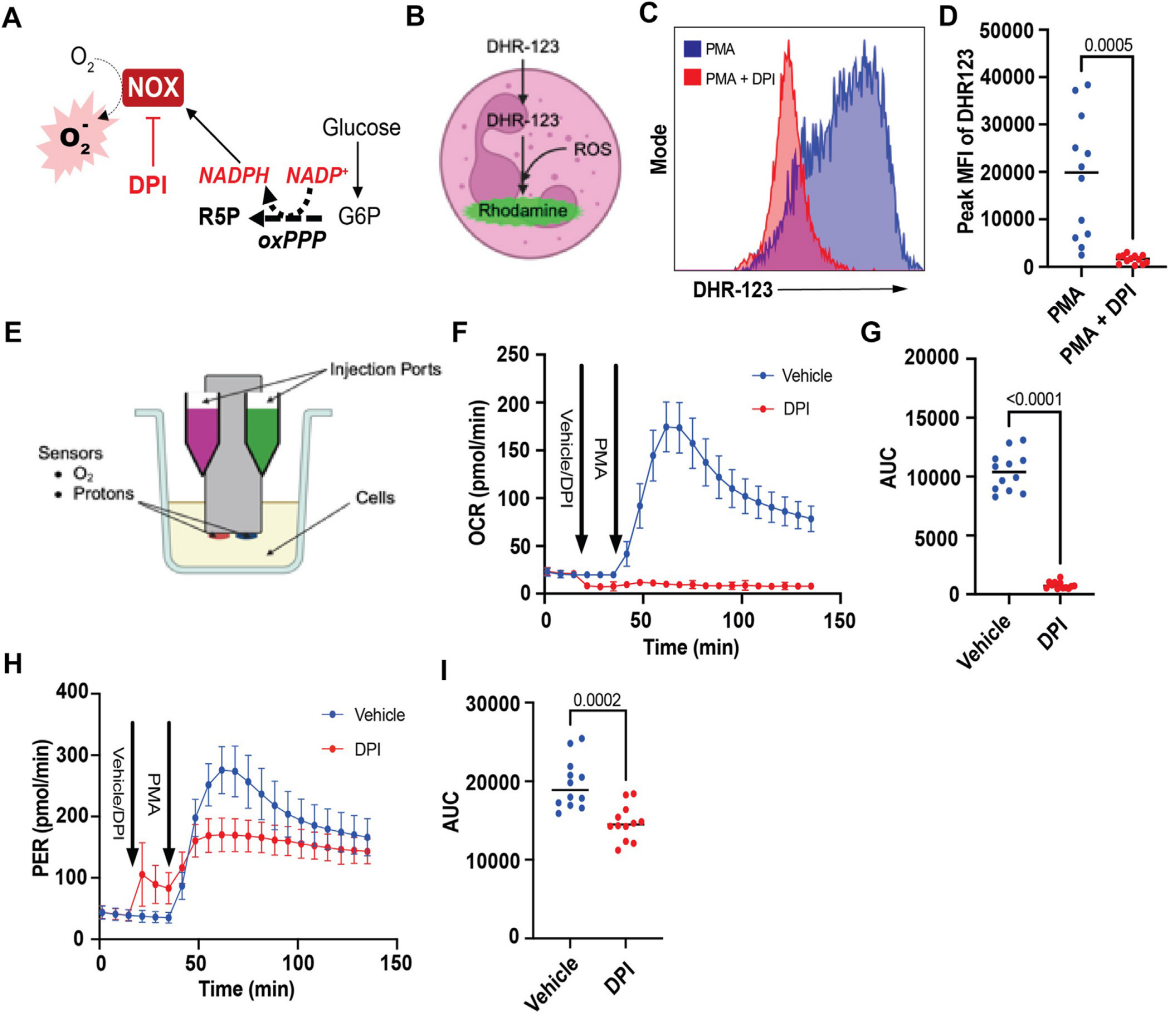
**Extracellular flux analysis provides temporal resolution of oxidative burst dynamics.** Measurement of reactive oxygen species (ROS) production in activated neutrophils: (*A*) schematic showing the known fundamental mechanism of the neutrophil respiratory burst. Upon stimulation, NADPH oxidase (Nox) promotes superoxide production, fueled by NADPH generated in the oxidative pentose phosphate pathway (oxPPP). Nox can be inhibited with diphenyleneiodonium (DPI, 10 μM) to assess methodologies for quantifying the respiratory burst. *B*, schematic showing dihydrorhodamine-123 (DHR-123) detection of ROS. *C*, flow cytometric detection of ROS production in neutrophils: Bone marrow–derived neutrophils from male and female mice were incubated with DHR-123 in the presence or absence of DPI (10 μM), followed by stimulation with phorbol 12-myristate 13-acetate (PMA; 500 nM) or vehicle (dimethyl sulfoxide). Flow cytometry was used to measure mean fluorescent intensity (MFI), as displayed in the representative histogram of DHR-123 fluorescence. *D*, peak MFI DHR-123 fluorescent signal. *E*, schematic showing sensors used to measure oxygen consumption and proton production, which can be used to measure ROS kinetics and acid extrusion in real time. *F*, examination of the temporal nature of the respiratory burst using extracellular flux analysis: bone marrow–derived neutrophils seeded into XFe96 plates were pretreated with vehicle of DPI *via* injection followed by injection of PMA and assessment of the oxygen consumption rate (OCR). *G*, area under the curve of OCR from *F*. *H*, parallel measurement of the proton efflux rate (PER) in PMA-treated cells in the presence or absence of DPI. *I*, PER area under the curve values. N = 12 mice/group (six males and six females/group). Statistical significance was evaluated by Student’s unpaired *t* test.

**Supplemental Fig. S1:**
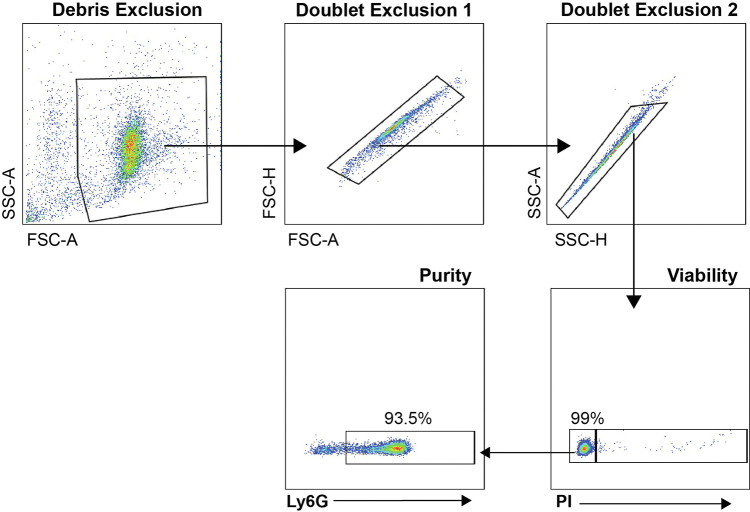


In recent years, extracellular flux analysis has been used to provide temporal and quantitative analyses of the neutrophil oxidative burst (11, 12, 13, 14). This method is advantageous because it provides a more resolved view of burst dynamics compared with classical fluorescent ROS indicators. To standardize methodology for measuring neutrophil-derived ROS, we first isolated neutrophils derived from murine bone marrow and examined their responses to phorbol 12-myristate 13-acetate (PMA), which initiates a robust neutrophil respiratory burst (15). As shown in Fig. S1, Ly6G and propidium iodide staining indicated that cells were 99% viable, and approximately 94% of these cells were neutrophils after magnetic bead sorting. To help ensure specificity, we pretreated neutrophils with diphenyleneiodonium (DPI), which inhibits Nox, preventing superoxide production and utilization of NADPH engendered by the oxPPP (Fig. 1*A*). Cumulative measurements of superoxide production using dihydrorhodamine-123 (DHR-123; Fig. 1*B*) indicated large increases in superoxide production in PMA-treated neutrophils, which were absent in neutrophils pretreated with DPI (Fig. 1, *C* and *D*).

To gain a more resolved view of respiratory burst kinetics, we seeded neutrophils into XFe96 tissue culture plates, promoted their adherence to the bottom of each well *via* mild centrifugation, and utilized XF sensor technology to measure oxygen consumption rate (OCR) and proton extrusion (*i.e.*, proton efflux rate [PER]) (Fig. 1*E*) (11, 12, 13, 14). After three baseline measurements, DPI or vehicle control was injected into each well, followed by three OCR measurements, injection of PMA, and subsequent temporal OCR measurements. DPI decreased the basal OCR, which is largely because of inhibition of flavoenzymes in the respiratory chain (16), indicating a low level of mitochondrial respiration in nonactivated neutrophils. Upon injection of PMA, the respiratory burst ensued, peaking ∼20 min after PMA injection and slowly tapering thereafter (Fig. 1*F*). Similar to findings with DHR-123, the presence of DPI completely inhibited the respiratory burst. Notably, area under the curve analysis indicated much less data variance compared with fluorometric measurements (Fig. 1, *D* and *G*). In addition, the XF method provides the extracellular acidification rate (ECAR), which relates to H^+^ extrusion caused by either glycolytic activity (17) or proton channels (18). As shown in Fig. 1*H*, ECAR dynamics, shown as the PER, matched the OCR data, confirming reports that H^+^ extrusion coincides with the respiratory burst (14). Although DPI significantly blunted the increase in glycolysis after PMA addition (Fig. 1*I*), DPI did not completely inhibit increases in PER after PMA addition, which suggests imperfect coupling of glycolytic rate with the respiratory burst.

### Neutrophil activation elevates levels of glycolysis-derived metabolites and decreases Krebs cycle metabolites

**Figure 2:**
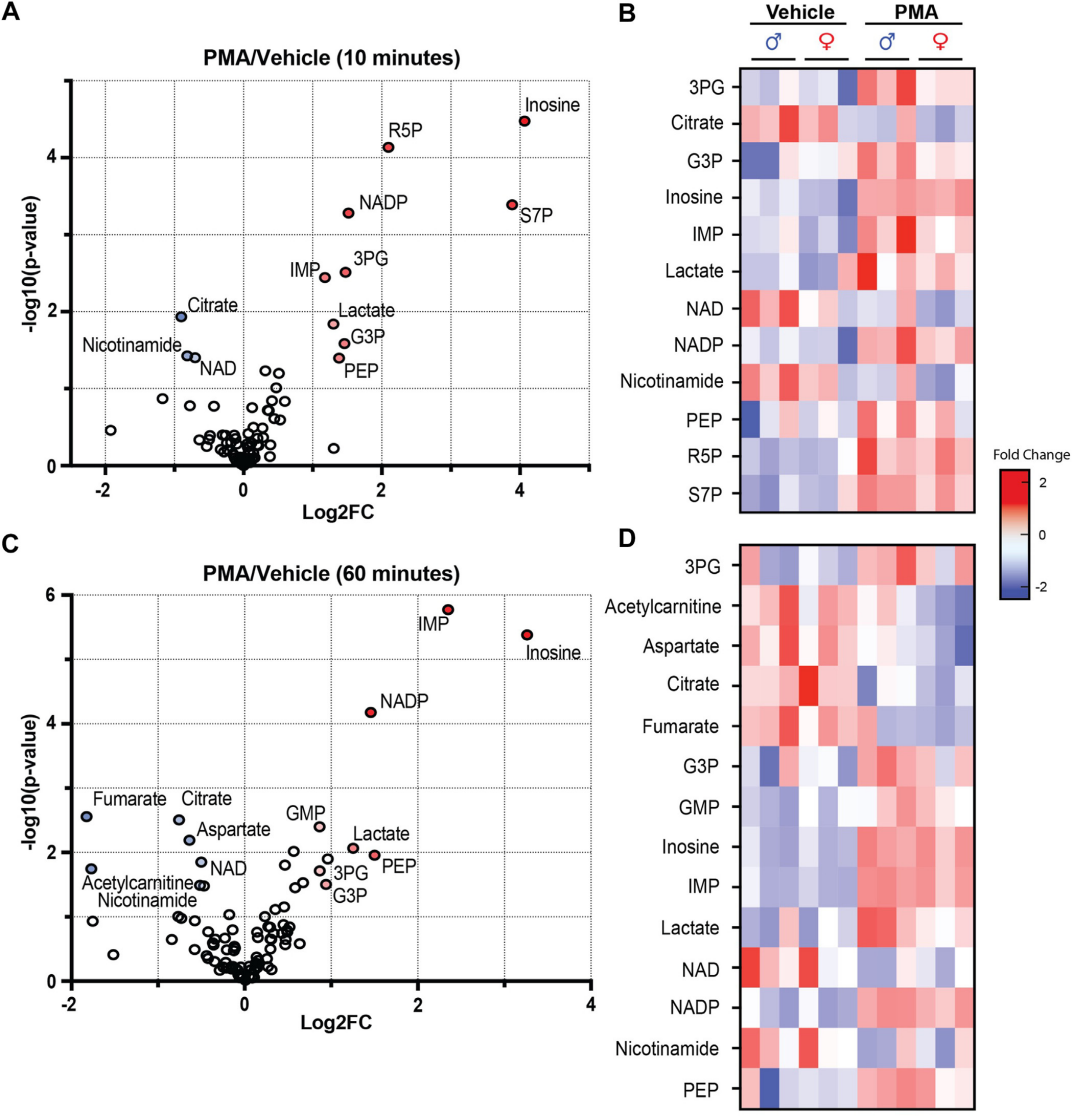
**Temporal changes in intermediary metabolite abundance in phorbol 12-myristate 13-acetate (PMA)-activated neutrophils.** Volcano plots and heatmaps of relative metabolite abundance in bone marrow–derived neutrophils activated with PMA (500 nM) or treated with vehicle control (dimethyl sulfoxide). *A* and *B*, metabolites that were significantly different after 10 min of PMA stimulation compared with control conditions. *C* and *D*, metabolites that were significantly different after 60 min of PMA stimulation. N = 6 mice/group (three males and three females/group). Metabolites that met a *p* value <0.05 were considered statistically significant (Student’s unpaired *t* test).

**Supplemental Fig. S2:**
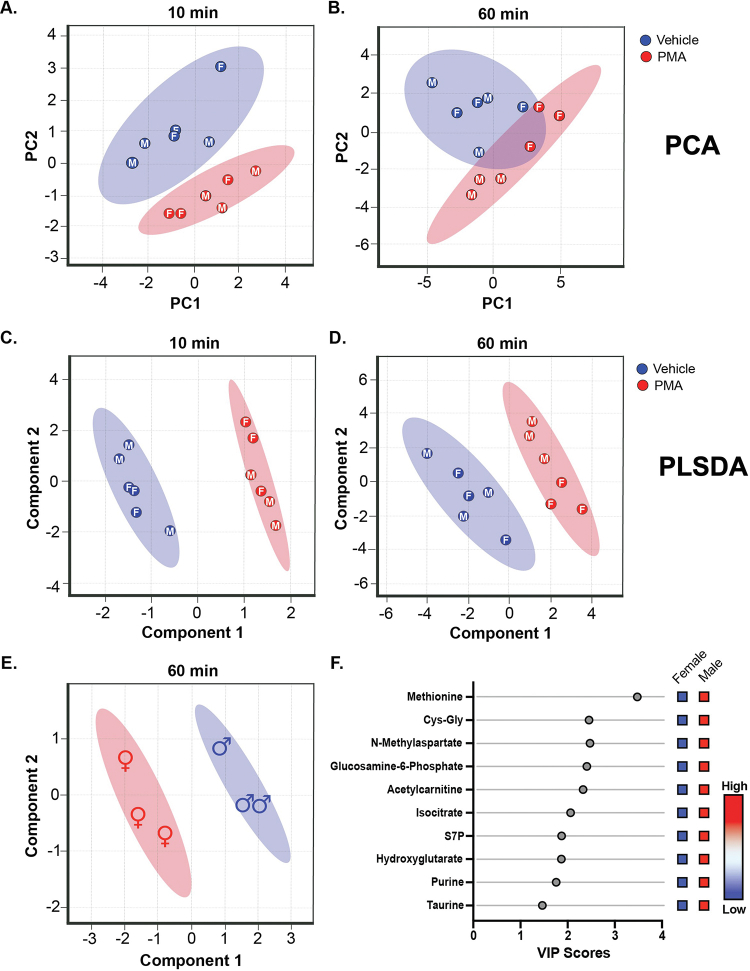


To establish a better understanding of how metabolic pathways change during the respiratory burst, we modeled the early and late respiratory burst by exposing neutrophils to PMA for 10 or 60 min. These times of the respiratory burst were chosen based on the time-dependent nature of the oxidative burst (Fig. 1), with 10 min being a temporal window before the burst has plateaued and 60 min being a window of the waning burst. After extraction of polar metabolites, we measured the relative abundance of 96 intermediary metabolites *via* mass spectrometry. After 10 min of PMA stimulation, we found elevated levels of PPP metabolites such as ribose 5-phosphate (R5P) and sedoheptulose 7-phosphate (S7P) as well as nucleosides and nucleotides such as inosine, inosine monophosphate, NADP^+^. Glycolytic intermediates including 3PG, phosphoenolpyruvate, and lactate were also elevated, as was the branchpoint intermediate glycerol 3-phosphate (G3P). Decreased in abundance were citrate, NAD^+^, and nicotinamide (Fig. 2*A*). Portrayed as a heatmap, the changes were found to be largely similar between male and female mice (Fig. 2*B*). After 60 min of PMA stimulation, inosine, inosine monophosphate, and NADP^+^ remained elevated; however, the PPP intermediates R5P and S7P were no longer significantly elevated (Fig. 2*C*). Three-carbon glycolytic intermediates and G3P remained higher through 60 min of the respiratory burst, and the Krebs cycle intermediates, citrate and fumarate, as well as the cataplerotic metabolite, aspartate, were notably lower in abundance. Although we found that the metabolic responses to acute PMA exposure appeared to be largely independent of biological sex (Figs. 2*D* and S2, *A* and *C*), we identified group separation between sexes after 60 min of PMA stimulation (Fig. S2, *B* and *D*), which variable importance in projection analyses indicated was due largely to increased levels of 10 metabolites in male compared with female mice (Fig. S2, *E* and *F*). These findings demonstrate rapid changes in the PPP, glycolysis, and mitochondrial metabolism that occur with neutrophil activation and highlight the possibility that ancillary pathways of glucose metabolism (*e.g.*, the G3P and serine biosynthesis pathways) may be important, unrecognized contributors to metabolic control of the respiratory burst.

### Stable isotope tracing shows activation of ancillary glucose metabolism pathways in activated neutrophils

**Figure 3:**
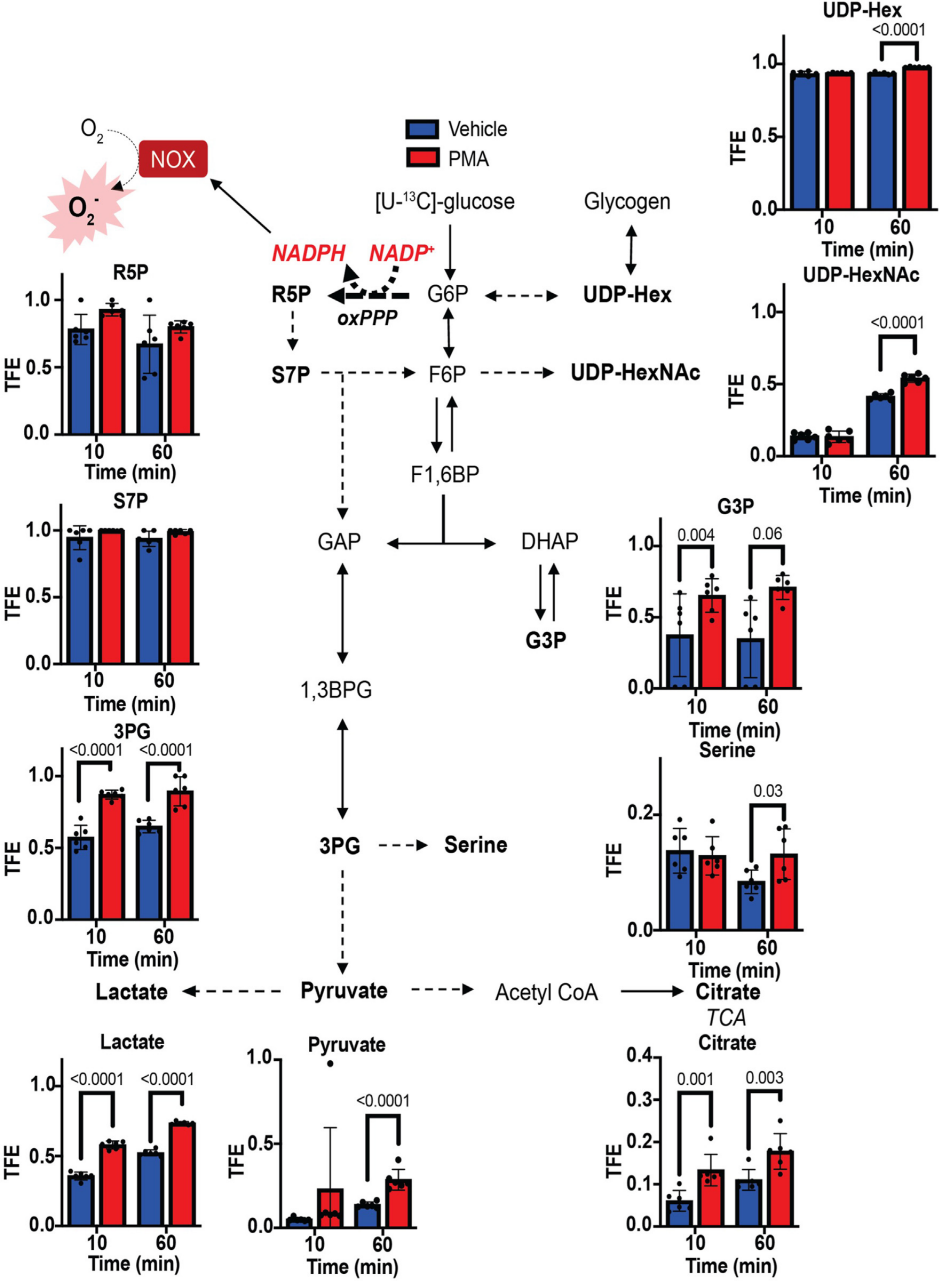
**Allocation of glucose-derived carbons to glycolytic intermediates and products of ancillary glucose pathways following neutrophil activation.** Bone marrow neutrophils from males and females were cultured with [U-^13^C]-glucose and activated with phorbol 12-myristate 13-acetate (500 nM) or vehicle (dimethyl sulfoxide) for 10 min or 60 min. Polar fractions were subjected to mass spectrometric analysis. Data shown are the total fractional enrichment (TFE) of glucose-derived ^13^C in metabolite pools. N = 6 mice/group (three males and three females/group). Data were transformed *via* logit transformation prior to Student’s unpaired *t* test.

For a more comprehensive understanding of carbohydrate metabolism, we utilized stable isotope metabolomics to measure incorporation of ^13^C-glucose-derived carbon into glycolytic intermediates and ancillary pathway products (19, 20). Neutrophils were activated with PMA for 10 or 60 min in the presence of ^13^C_6_-glucose, after which metabolites were extracted and examined *via* mass spectrometry. By examining total fractional enrichment, we found that the PPP and the glycogen synthesis/breakdown pathway carried high flux even basally. The PPP metabolites, R5P and S7P, were highly enriched with ^13^C even in the vehicle control, with total fractional enrichment nearing 100% with PMA stimulation (Fig. 3). Similarly, the glycogen intermediate UDP-hexose (UDP-Hex) was more than 90% labeled in control cells; however, further enrichment in UDP-Hex was achieved after 60 min of PMA stimulation, suggestive of phasic regulation of glycogen metabolism. Interestingly, the ^13^C-labeled uridine diphosphate *N*-acetylhexosamine pool provided evidence that the hexosamine biosynthetic pathway is activated only during the later phase of the respiratory burst, with markedly higher labeling after 60 min of PMA stimulation compared with 10 min of stimulation.

The labeling of glycolytic intermediates and ancillary metabolites also indicated activation of glycolysis and pathways stemming from the 3-carbon glycolytic pool. Approximately 50 to 60% of the 3PG pool was enriched with ^13^C in control neutrophils, and, with PMA stimulation, reached a steady state of ∼80% ^13^C enrichment. Similarly, ^13^C enrichment of lactate and pyruvate was markedly increased after PMA stimulation. Ancillary pathways branching from DHAP and 3PG also appeared to carry higher flux after neutrophil activation. The G3P pool was more enriched in activated neutrophils, with ^13^C labeling reaching steady state within 10 min of PMA stimulation. Furthermore, serine was 10 to 20% labeled, regardless of activation state; yet, compared with corresponding nonactivated neutrophils, serine from PMA-stimulated cells showed higher levels of ^13^C label after 60 min. The fact that basal ^13^C labeling of the serine pool diminished by 60 min of labeling suggests dilution of the pool by nonlabeled substrates (Fig. 3). Interestingly, PMA stimulation also increased labeling of citrate at both the early and later stages of the respiratory burst, indicating that neutrophil activation generally augments glucose oxidation. Collectively, these data provide evidence of a coordinated metabolic program triggered in activated neutrophils that is characterized by augmented glycolysis, increased utilization of ancillary pathways of glucose metabolism, and higher mitochondrial metabolism.

### Glycogen fuels the early oxidative burst in activated neutrophils

**Figure 4:**
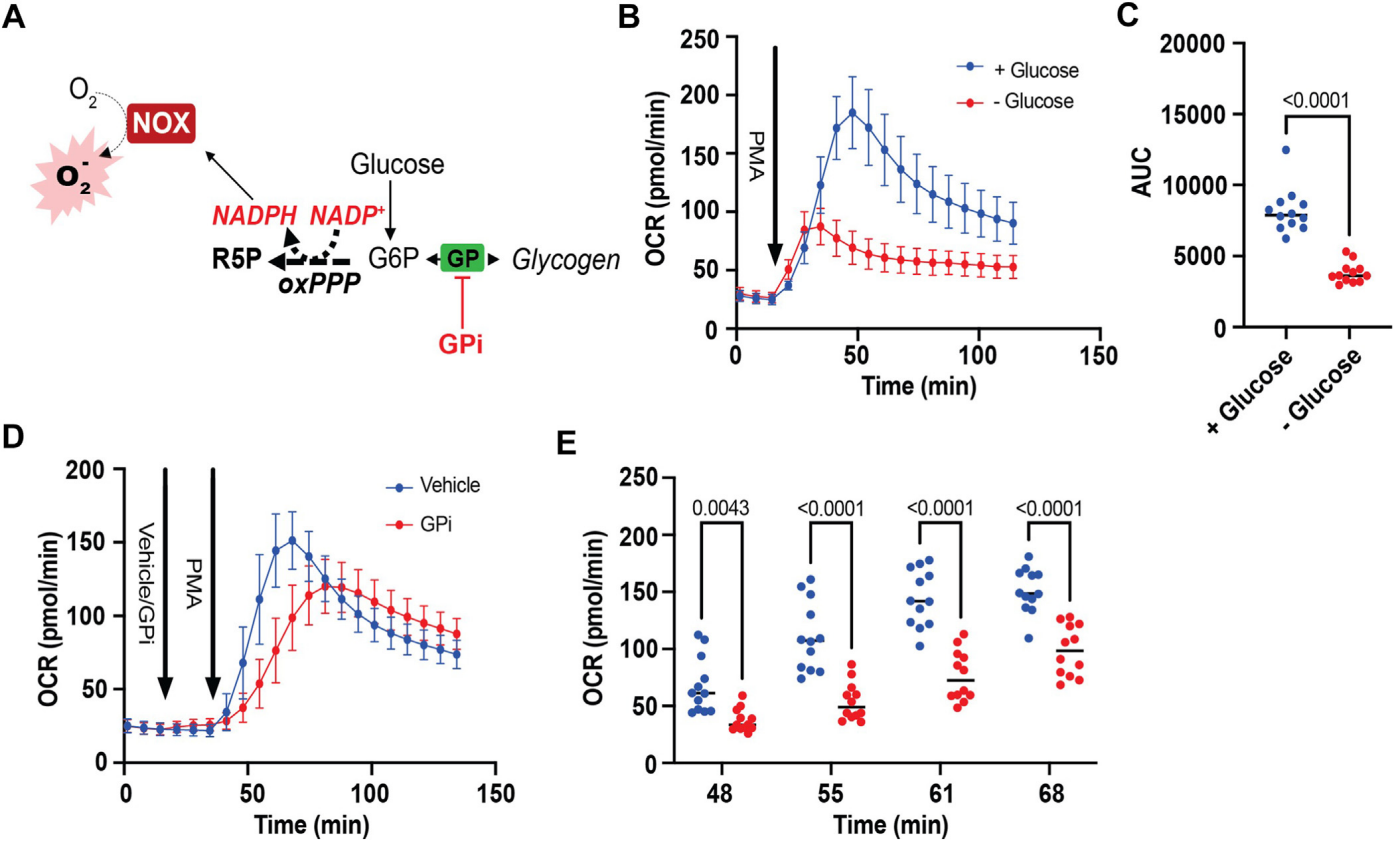
**Glycogen metabolism supports the early oxidative burst in neutrophils.** Measurement of the respiratory burst in murine bone marrow–derived neutrophils in the presence or absence of glucose or with glycogen phosphorylase inhibition: (*A*) schematic showing the potential of glycogen to contribute to the respiratory burst. *B*, oxygen consumption rate (OCR) trace of neutrophils incubated in glucose-replete or glucose-free media and stimulated with phorbol 12-myristate 13-acetate (500 nM). *C*, area under the curve analysis of the OCR trace in *B*. N = 12 (six males and six females/group). Statistical significance was assessed *via* Student’s *t* test. *D*, OCR trace of neutrophils in glucose-replete media with injection of glycogen phosphorylase inhibitor (GPi, 100 μM) or vehicle (dimethyl sulfoxide). *E*, OCR readings during the first 20 min of the respiratory burst. N = 12 (six males and six females/group). Statistical significance was assessed by repeated-measures ANOVA with Tukey post-test.

Because the respiratory burst requires 6-carbon glycolytic intermediates to fuel NADPH generation through the oxPPP (8), we performed negative control experiments in which neutrophils were deprived of glucose or were in glucose-replete conditions (Fig. 4*A*). As shown in Figure 4, *B* and *C*, baseline OCR was identical in the absence or presence of glucose, and, upon addition of PMA, there was a stark decrease in the respiratory burst under glucose-limited conditions. Nevertheless, the initial 15 min of the oxidative burst showed nearly identical kinetics in the absence or presence of glucose, indicating that glucose derived from intracellular sources is likely used to fuel the respiratory burst. Given that glycogen is the prototypical storage depot for glucose and has been shown to influence neutrophil effector functions (21), we next tested whether, in the presence of glucose, inhibition of glycogen breakdown using glycogen phosphorylase inhibitor (GPi) alters respiratory burst dynamics. As shown in Figure 4*D*, neutrophils pretreated with GPi showed delayed ROS kinetics, insinuating that glycogen breakdown supplies G6P to fuel the initial respiratory burst. Time-dependent assessments of the OCR readings showed statistically lower ROS production in GPi-treated cells, which began only a few minutes after PMA addition and lasted for ∼20 min (Fig. 4*E*).

**Supplemental Fig. S3:**
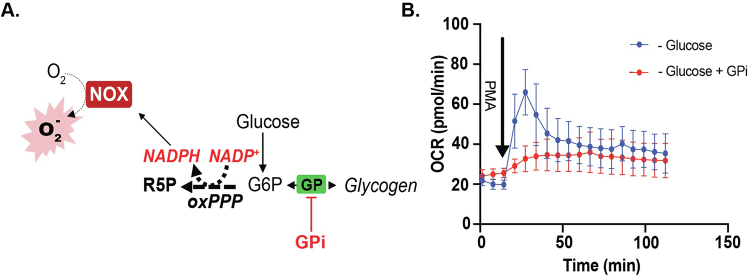


To further delineate the impact of glycogen catabolism on the oxidative burst, we pretreated neutrophils with vehicle or GPi in a glucose-free environment and then measured the respiratory burst upon PMA injection (Fig. S3*A*). In the absence of glucose, we observed an early oxidative burst that dissipated within 30 min. Consistent with glycogen fueling of this early burst, the presence of GPi completely abolished the increase in OCR caused by PMA treatment (Fig. S3*B*). Collectively, these results indicate that neutrophils have a phase-partitioned respiratory burst, with glycogen breakdown contributing solely to the initial respiratory burst, followed by burst support *via* catabolism of extracellular glucose.

### Inhibition of the serine biosynthesis pathway extends the respiratory burst

Although the oxPPP is the largest source of NADPH regeneration in most cells, we ventured to determine if other potential NADPH-regenerating pathways might contribute to the respiratory burst. An alternative pathway could be serine-driven one-carbon metabolism, in which methylene tetrahydrofolate oxidation to 10-formyl tetrahydrofolate is coupled to reduction of NADP^+^ to NADPH (22). Thus, it is possible that *de novo* serine biosynthesis in neutrophils could provide NADPH for the respiratory burst. Furthermore, our stable isotope metabolomics data indicated higher ^13^C enrichment in serine after 60 min of PMA stimulation. Hence, a competing hypothesis is that the serine biosynthetic pathway (SBP) could modulate the respiratory burst *via* a “carbon stealing” mechanism, which may utilize glucose-derived carbon that could otherwise be allocated to the oxPPP. Consistency with the former hypothesis would be suggested by lower ROS production upon inhibition of the SBP, whereas consistency with the “carbon stealing” hypothesis would be suggested by higher ROS production with SBP inhibition.

**Figure 5:**
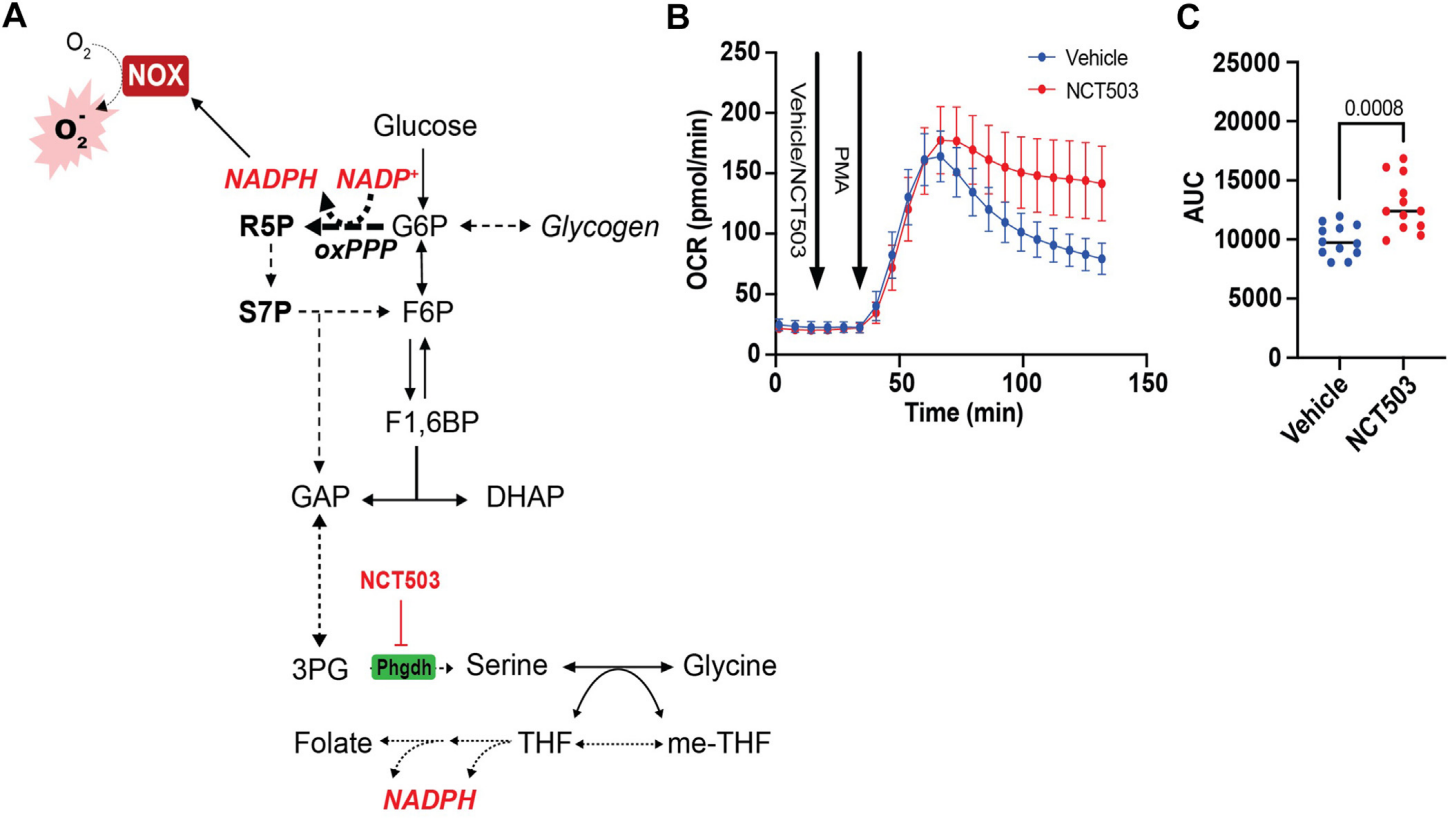
**The serine biosynthesis pathway restricts the late phase of the neutrophil oxidative burst.** Measurement of the respiratory burst in murine bone marrow–derived neutrophils in the presence or absence of phosphoglycerate dehydrogenase (Phgdh) inhibitor: (*A*) schematic depicting integration of the serine biosynthesis pathway with metabolic pathways important for neutrophil metabolism. *B*, the respiratory burst in bone marrow–derived neutrophils incubated with the Phgdh inhibitor NCT503 (20 μM) or vehicle (dimethyl sulfoxide) and activated with phorbol 12-myristate 13-acetate (500 nM). *C*, area under the curve analysis of the OCR trace in *B*. N = 12 (six males and six females/group). Significance was assessed by Student’s unpaired *t* test. OCR, oxygen consumption rate.

To address this experimentally, we examined whether inhibition of the committed step of serine biosynthesis, that is, 3-phosphoglycerate dehydrogenase (Phgdh), with NCT503 influences the respiratory burst (Fig. 5*A*). As shown in Figure 5*B*, Phgdh inhibition had no effect on basal OCR or the initial respiratory burst; however, it prolonged the burst significantly. Area under the curve analysis indicated that NCT503-induced prolongation of the respiratory burst led to significantly more ROS generation (Fig. 5*C*). Taken together, these findings indicate that the activity of the SBP contributes to the duration of the respiratory burst, which appears consistent with a carbon stealing mechanism that functions as a late restraint mechanism to control the respiratory burst.

### Inhibition of the payoff phase of glycolysis does not affect the respiratory burst

**Supplemental Fig. S4:**
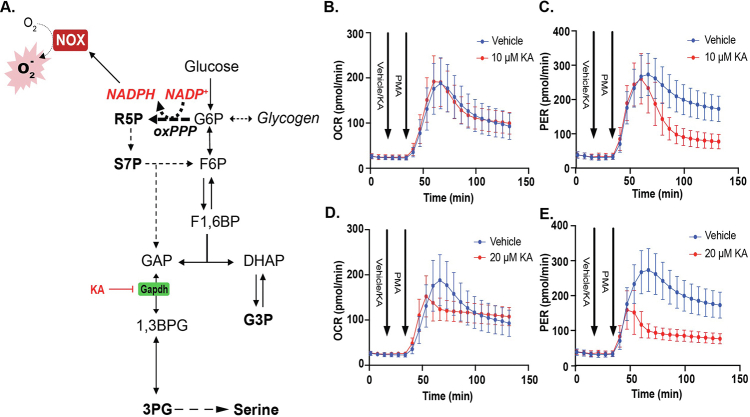


The finding that inhibition of the SBP augmented ROS during the respiratory burst could suggest that ancillary biosynthetic pathways could act as carbon sinks that siphon glucose-derived carbon, leaving less available for entry into the oxPPP or other fates. The corollary of this hypothesis is an extension of crossover theorem (23, 24, 25, 26) and suggests that inhibition of glycolysis downstream of the phosphofructokinase step would decrease glucose catabolism, leaving more glucose-derived carbon available for oxPPP entry, which would fuel the respiratory burst. To test this possibility, we used koningic acid (KA) to inhibit GAPDH, which would be expected to diminish glycolytic ATP production in the payoff phase of glycolysis (Fig. S4*A*). Thus, we pretreated neutrophils with 0 to 20 μM KA and assessed its effects on PMA-induced changes in PER and OCR. Neutrophils exhibited a concentration-dependent decrease in PER, reflective of decreased glycolytic activity (Fig. S4, *B–D*). Surprisingly, however, we found no significant decreases in the respiratory burst, even at the highest concentrations of KA (Fig. S4, *B–G*). These findings appear to indicate that the respiratory burst can continue even when glycolytic ATP production is inhibited, which suggests that alternative pathways of ATP production likely contribute to ATP levels in neutrophils during the respiratory burst.

### The G3P shuttle contributes to the amplitude of the oxidative burst

**Figure 6:**
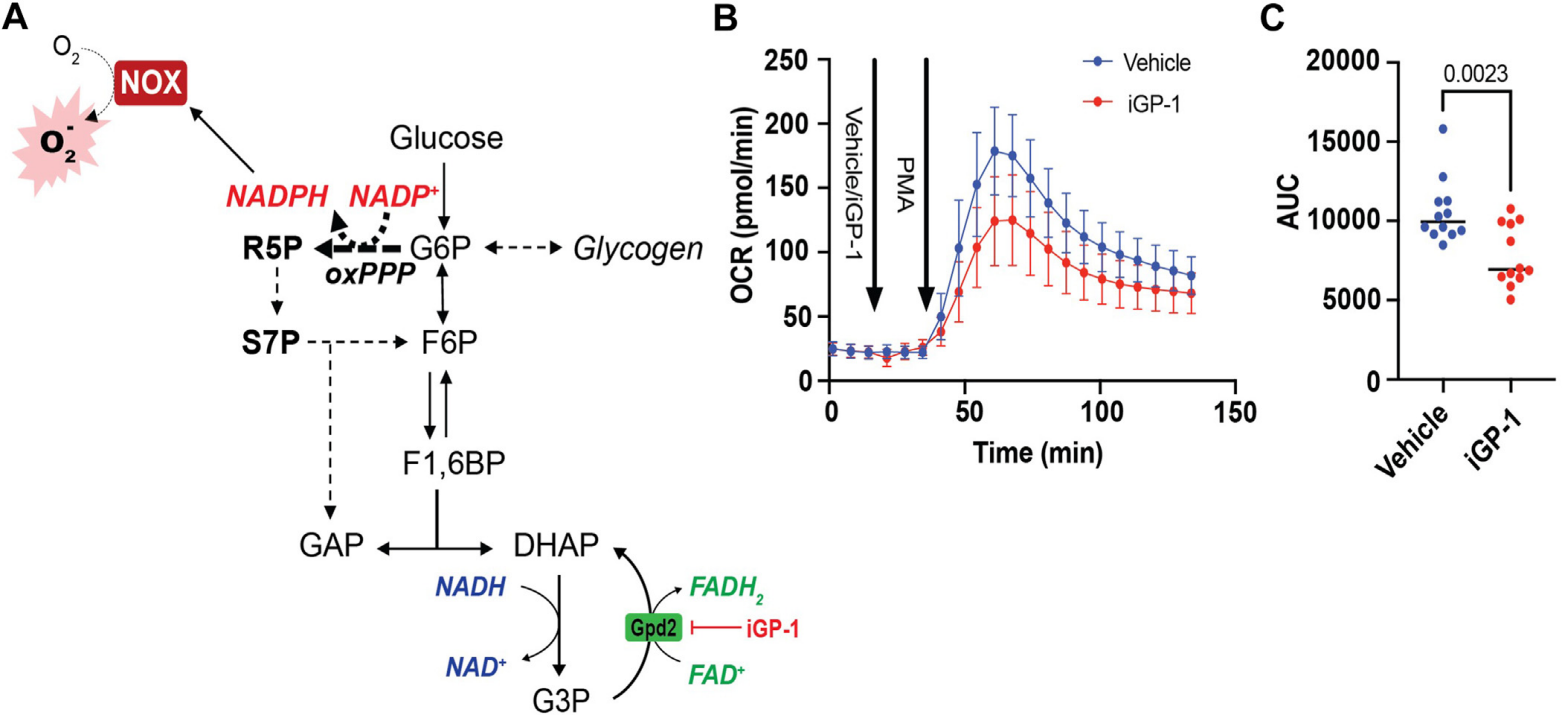
**The glycerol 3-phosphate (G3P) shuttle supports the neutrophil oxidative burst.** Measurement of the respiratory burst in murine bone marrow–derived neutrophils in the presence or absence of G3P shuttle inhibitor: (*A*) schematic depicting integration of the G3P shuttle with metabolic pathways important for neutrophil metabolism. *B*, measurement of the respiratory burst of bone marrow–derived neutrophils incubated with Gpd2 inhibitor (iGP-1, 1 mM) or vehicle (dimethyl sulfoxide). *C*, area under the curve analysis of the OCR trace in *B*. N = 12 (six males and six females/group). Significance was assessed by Student’s unpaired *t* test. OCR, oxygen consumption rate.

Given that PMA treatment led to rapid channeling of glucose-derived carbon to form G3P, and because the G3P shuttle has been suggested to contribute to mitochondrial ROS production (27), we next questioned whether the G3P shuttle influences the respiratory burst. Therefore, we pretreated neutrophils with the glycerol 3-phosphate dehydrogenase 2 (Gpd2) inhibitor, iGP1, and examined its effect on the respiratory burst (Fig. 6*A*). Of note, inhibition of Gpd2 would inhibit the G3P shuttle by preventing cycling of G3P back to DHAP and simultaneously preventing offloading of electrons to FAD^+^, with the reduced FAD pool (FADH_2_) used to fuel mitochondrial respiration; it would also likely decrease NAD^+^ regeneration (27). As shown in Figure 6, *B* and *C*, Gpd2 inhibition decreased the respiratory burst by ∼30%, suggesting that the NAD^+^ regenerative activity of the G3P shuttle, or its ability to deposit electrons in the electron transport chain, is a critical determinant of the neutrophil oxidative burst.

### Inhibition of mitochondrial complex III regulates the late phase of the respiratory burst

**Figure 7:**
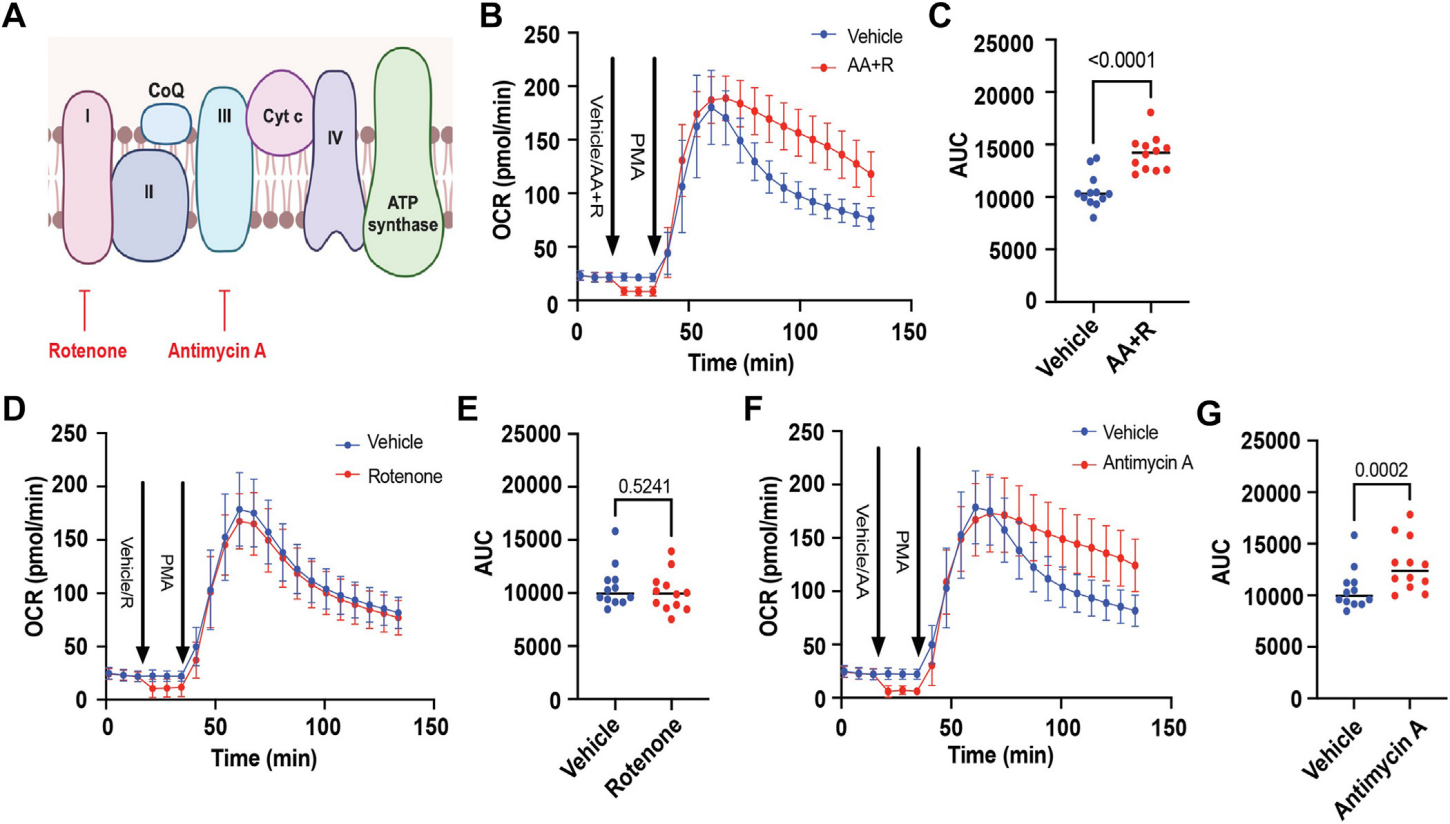
**Inhibition of mitochondrial complex III prolongs the neutrophil respiratory burst.** Measurement of the respiratory burst in murine bone marrow–derived neutrophils in the presence or absence of mitochondrial respiratory complex inhibitors: (*A*) schematic depicting sites of inhibition by the complex I inhibitor, rotenone, and the complex III inhibitor, antimycin A (AA). *B*, measurement of the respiratory burst of bone marrow–derived neutrophils incubated with AA (1 μM) and rotenone (0.5 μM) or vehicle (dimethyl sulfoxide [DMSO]) followed by PMA stimulation (500 nM). *C*, area under the curve analysis of the OCR trace in *B*. *D*, measurement of the respiratory burst of bone marrow–derived neutrophils incubated with rotenone alone (0.5 μM) or vehicle (DMSO) followed by PMA stimulation (500 nM). *E*, area under the curve analysis of the OCR trace in *D*. *F*, measurement of the respiratory burst of bone marrow–derived neutrophils incubated with AA alone (1 μM) or vehicle (DMSO) followed by PMA stimulation (500 nM). *G*, area under the curve analysis of the OCR trace in *F*. N = 12 (six males and six females/group). Significance was assessed by Student’s unpaired *t* test. OCR, oxygen consumption rate; PMA, phorbol 12-myristate 13-acetate.

The finding that neutrophil activation increases glucose-derived carbon allocation into citrate (Fig. 3) and that the G3P shuttle is activated by PMA insinuates a potential role of mitochondria in the respiratory burst. To determine whether inhibition of respiration influences neutrophil ROS production, we first completely blocked mitochondrial electron transport by simultaneously inhibiting complexes I and III using rotenone and antimycin A (AA), respectively (Fig. 7*A*). Upon injection of these inhibitors, we observed an immediate decrease in basal OCR, which represents residual mitochondrial activity in unstimulated neutrophils (Fig. 7*B*). Upon PMA injection, we found that neutrophils with blocked electron transport showed a prolonged respiratory burst (Fig. 7, *B* and *C*). To determine which respiratory complex may be responsible for this effect, we next inhibited complex I or III individually. As shown in Figure 7, *D* and *E*, rotenone inhibited basal OCR, suggesting that residual mitochondrial activity in unstimulated neutrophils is fueled at least in part by electron entry into complex I; however, upon PMA activation, rotenone had no effect on the respiratory burst. As expected, inhibition of complex III with AA also decreased basal OCR; however, in contrast to rotenone, AA significantly prolonged ROS production in PMA-stimulated neutrophils (Fig. 7, *F* and *G*). These findings indicate that complex III of the respiratory chain contributes significantly to the phasic nature of the neutrophil respiratory burst.

## Discussion

Recent studies have shown that neutrophil function is exquisitely dependent on their metabolism, with increasing appreciation for how pathways other than the oxPPP control neutrophil responses (6). In particular, glycogenolysis (9, 10), the G3P shuttle (27), and mitochondrial activity (28) have been found to influence ROS production or other essential neutrophil functions. Nevertheless, how these and other metabolic pathways shape the functional responses of neutrophils after their activation remains unclear. To address this gap in knowledge, we exploited extracellular flux technology and stable isotope metabolomics to understand how metabolic pathway activity changes with activation of the neutrophil respiratory burst. Targeted inhibition of metabolic pathways found to change in activated neutrophils revealed how ancillary pathways of glucose metabolism and mitochondrial activity influence ROS production. We found that, in addition to the PPP, the glycogenolysis/biosynthesis pathway carries high flux, and inhibition of glycogen breakdown prevents the initial phase of the respiratory burst. Moreover, our data provide evidence that neutrophil stimulation activates the G3P shuttle and serine synthesis, which were found to contribute to the amplitude and length, respectively, of the respiratory burst. We also found that inhibition of mitochondrial complex III influences the duration of neutrophil ROS generation. Taken together, these findings provide new resolution of neutrophil dynamics and suggest that ancillary pathways of glucose metabolism and mitochondrial activity play key roles in the initiation, magnitude, and duration of the respiratory burst.

Our findings contribute to the growing body of knowledge implicating glycogen metabolism in immune cell biology. In human neutrophils, glycogen was found to be a major metabolic source, contributing to nearly 50% of the glycolytic intermediate pool (9). Furthermore, inhibition of glycogenolysis increased neutrophil migration (9), whereas deletion of glycogen synthase diminished bacterial killing (10). We extend understanding of the role of glycogen in neutrophil function by showing that glycogen appears solely responsible for the initial phase of the respiratory burst. Experiments in which we cultured murine neutrophils in glucose-replete or -free conditions in the absence or presence of GPi demonstrated that exogenous glucose is not necessary for the first 15 to 20 min of the respiratory burst. Stable isotope labeling experiments showed that within 10 min, even in nonstimulated conditions, the UDP-Hex glycogen precursor pool is ∼90% labeled, with labeling increasing further after 60 min of PMA stimulation. These findings suggest that the glycogen synthesis pathway carries high flux, perhaps paralleling that of the oxPPP, and that glycogen is critical for priming and initiating the respiratory burst.

Given that previous studies demonstrate that, despite its increased utilization, glycogen levels do not fall and may even increase upon neutrophil activation (9, 10), it is likely that neutrophils facilitate rapid glycogen cycling that both increases glycogen synthesis and utilization. Such constant use and replenishment of the glycogen pool may provide flexibility to neutrophils under hypoxic, low glucose environments. Moreover, glycogen appears to be important for NETosis, at least in the context of coronavirus disease 19 infection (29), and glycogen storage diseases have long been known to promote neutropenia and decrease the respiratory burst and impair neutrophil trafficking, phagocytosis, and bacterial killing (30, 31, 32, 33). Given dynamic relationships of glycogen depots with nutritional state, it is tempting to speculate that conditions that lead to glucose dysregulation, for example, diabetes, may promote immune dysfunction by altering glycogen dynamics in neutrophils. Indeed, metabolic reprogramming in diabetic neutrophils appears to be at least partially causative of the higher levels of infection in diabetic patients; interestingly, diabetic neutrophils demonstrate activated polyol and hexosamine biosynthetic pathways (34, 35, 36, 37), which could compete with the oxPPP and the glycogen pathway for glucose-derived carbon.

Although glycogen was found to contribute to the initial respiratory burst, exogenous glucose was required to power the middle and later phases of the oxidative burst. Our findings insinuate that the G3P synthesis pathway may contribute to how glucose is utilized to control the magnitude of ROS production. We observed a near doubling of the ^13^C label from ^13^C_6_-glucose in the G3P pool after PMA addition and found that inhibition of GPD2—the mitochondrial isoform of G3P dehydrogenase that can facilitate electron transfer to the ubiquinone pool and complex III—significantly diminished the magnitude of the respiratory burst. This is interesting given that mitochondria within neutrophils maintain a membrane potential, with G3P supporting the highest mitochondrial membrane potential (38). It has been posited that mitochondrial activity facilitated by the G3P shuttle maintains neutrophil viability (6), which may be responsible for maintaining energy levels under conditions in which glycolytic ATP production is insufficient. We speculate that this mechanism may be responsible for maintaining neutrophil function during the respiratory burst when the GAPDH inhibitor, KA, was present (Fig. S4). Previous studies also indicate that the G3P shuttle contributes to mitochondrial ROS (27). Because our assays do not discriminate Nox-derived ROS from mitochondrial ROS, it remains possible that a portion of the respiratory burst may derive from mitochondrially generated superoxide.

In this respect, perhaps the most difficult interpretation derives from our experimental results showing that inhibition of complex III prolongs the respiratory burst. Taken at face value, the data suggest that mitochondrial activity normally contributes to oxidative burst decay; however, inhibition of complex III with AA may also increase mitochondrial ROS under some conditions (39, 40, 41). Interestingly, in mitochondria from multiple tissue types, the G3P shuttle regulates mitochondrial ROS production, particularly when complex III is inhibited by AA (42). Thus, it is possible that the prolonged respiratory burst occurring with AA is due to a switch from Nox-derived ROS to mitochondrial ROS. Given that AA (as well as many other mitochondrial poisons) are metabolites produced by bacteria such as *Streptomyces* (43), it is plausible that the heightened ROS production of neutrophils in the presence of AA is an evolutionarily conserved mammalian mechanism to combat pathogens. Nevertheless, it has been shown that inhibition of complex III impairs neutrophil antimicrobial activity and NETosis and that mitochondrial ROS scavenging with MitoTEMPOL attenuates bacterial killing (44). Further studies will be required to delineate clearly the impact of the G3P shuttle and complex III-related mitochondrial activity in the numerous facets of neutrophil antimicrobial activity.

Our study also revealed other pathways, for example, the serine biosynthesis pathway, that could contribute to the duration of the respiratory burst. Guided by observations that show the importance of the serine biosynthesis pathway and folate cycle in NADPH regeneration, we initially posited that this pathway may supplement the NADPH pool to fuel the respiratory burst. However, our results appear to rule out this possibility, given that inhibition of the committed step of serine biosynthesis, that is, Phgdh, prolonged the respiratory burst. Doubt of the validity of this hypothesis is further afforded by the fact that NADPH from the folate cycle is generated in mitochondria, which would impart a compartmental hurdle for delivery of mitochondrial NADPH to the cytosolic pool. Rather, our data are more consistent with a “carbon stealing” mechanism that diminishes the availability of carbon for eventual entry into the oxPPP. Although the branchpoint at which carbon enters the serine biosynthesis pathway is many enzymatic steps away from the oxPPP, neutrophils have been shown to leverage gluconeogenesis for effective bacterial killing (10). Thus, it remains possible that levels of the 3-carbon glycolytic intermediate pool, which could be affected by SBP activity, are critical for providing substrate to gluconeogenic enzymes such as fructose bisphosphatase to supplement levels of G6P to fuel the respiratory burst. The fact that ^13^C enrichment of serine was significantly elevated only in the late stages of the respiratory burst is also consistent with the notion that the serine biosynthesis pathway controls the duration of the oxidative burst.

Although our findings contribute to understanding of the role of metabolic accessory pathways and mitochondria in the phasic respiratory burst, several limitations should be noted. Our study focused solely on how metabolism regulates neutrophil ROS generation and did not address the many other facets of neutrophil function, such as chemotaxis, phagocytosis, and NETosis. Studies that address the role of each accessory pathway and mitochondrial activity on these neutrophil functions will likely reveal new understanding of how metabolism coordinates pathogenic responses. Moreover, we did not address how the hexosamine biosynthetic pathway, which was activated in the late stage of the respiratory burst, influences ROS production. Although inhibitors for this pathway exist, they are notorious for being rather nonspecific. Thus, genetic interventions targeting the hexosamine pathway would be required to delineate its significance to neutrophil antimicrobial activity. Also, we did not address how substrates other than glucose impact the respiratory burst, and it remains possible that fatty acids, ketone bodies, and amino acids contribute significantly to glucose carbon handling and the respiratory burst. Last, additional experiments will be required to determine if the findings that occur in primary neutrophils *in vitro* are consistent *in vivo* and whether the findings in mice are consistent with that which occurs in humans.

In summary, our findings demonstrate that the respiratory burst of neutrophils is partitioned into phases that are deftly controlled by accessory pathways of glucose metabolism and mitochondrial activity. We show that glycogen catabolism fuels the initial phase of the oxidative burst, that the G3P shuttle influences the magnitude of the burst, and that the serine biosynthesis pathway and mitochondrial complex III control the duration of ROS production. An exciting prospect for future studies is to understand how these and other metabolic pathways control the health and function of the immune system under conditions of normal physiological stress and disease.

## Experimental procedures

### Animal models

All animal procedures were performed following the Guide for the Care and Use of Laboratory Animals published by the US National Institutes of Health and were approved by the University of Louisville Institutional Animal Care and Use Committee. Male and female C57BL/6J mice (14–22 weeks of age) were purchased from Jackson Laboratories and housed in a pathogen-free facility under a standard 12 h light–dark cycle with ad libitum access to food and water.

### Neutrophil isolation

Bone marrow cells were isolated by flushing cold MACS Rinsing Solution supplemented with 0.5% Bovine Serum Albumin Stock Solution (Miltenyi Biotech) through murine femurs and tibias with a 21-gauge needle. Bone marrow neutrophils were isolated with a neutrophil isolation kit (Miltenyi Biotech), according to the manufacturer’s recommendations. Briefly, neutrophils were isolated by depletion of nontarget cells. Nontarget cells were labeled with a cocktail of biotin-conjugated monoclonal antibodies, as primary labeling reagent, followed by antibiotin monoclonal antibodies conjugated to MicroBeads as a secondary labeling reagent. The magnetically labeled nontarget cells were depleted by retaining them on LS MACS Column in the magnetic field of a MACS separator, while unlabeled neutrophils ran through the column. Neutrophil purity and viability were evaluated by Ly6G expression and propidium iodide staining with flow cytometry, as described (45, 46).

### ROS measurement with DHR-123

Neutrophils (2 × 10^6^ cells/ml) suspended in Dulbecco's modified Eagle's medium (DMEM) supplemented with 5.5 mM glucose, 4 mM glutamine, and 1 mM pyruvate were allowed to equilibrate for 15 min in a 37 °C aluminum bead bath. Following equilibration, 1 ml of the aforementioned media containing 1 μg/ml DHR-123 (Cayman; catalog no. 85100) and 5 μM PMA was added to 1 ml of the cell suspension. This brought the final concentration of DHR-123 to 0.5 μg/ml and the concentration of PMA (Sigma–Aldrich; catalog no.: P1585) to 500 nM. After a 30 min incubation, the cells were washed once in 1× PBS (GIBCO) and were then suspended in 1 ml of PBS and analyzed *via* an LSRFortessa X-20. Data analysis was performed out using FlowJo, v10.8 (BD Biosciences).

### Extracellular flux analysis

Neutrophils were seeded onto Seahorse XFe96 Cell Culture Plates (Agilent), as described (11, 13, 14). Briefly, XF96 culture plates were coated with 25 μl of Poly-l-Lysine (Sigma Life Science) for 5 min, washed with sterile water, and allowed to dry for 2 h. Neutrophils were seeded and adhered to the plate by pipetting 50 μl containing 1 × 10^5^ cells into each well and centrifuging at 200*g* for 1 min with no braking. The plate was incubated at 37 °C for 25 min in a non-CO_2_ incubator, then 130 μl of Seahorse XF DMEM, pH 7.4, supplemented with 5.5 mM glucose, 4 mM glutamine, and 1 mM pyruvate (unless otherwise specified) (Agilent) was added for a final volume of 180 μl. After 15 min of incubation, plates were loaded into the XFe96 analyzer (Agilent). After three baseline readings, pharmacological agents were injected *via* port A, as indicated in the text; these inhibitors included DPI (Cayman), GPi (Cayman), KA (Cayman), phosphoglycerate dehydrogenase inhibitor (NCT503; Sigma–Aldrich), Gpd2 inhibitor (iGP-1; Focus Biomolecules), and mitochondrial complex inhibitors (AA, rotenone [R]; Sigma–Aldrich). Following three more recordings, PMA (500 nM, final) was injected to initiate the oxidative burst. The OCR and ECAR were measured for up to approximately 120 min.

### Stable isotope resolved metabolomics

Analysis of the fate of glucose was performed, essentially as described (19, 20, 47, 48). Neutrophils (5 × 10^6^/ml) were incubated at 37 °C in DMEM with either universally labeled glucose (^13^C_6_-glucose; Cambridge Isotope Laboratories) or unlabeled glucose (5.5 mM) in the presence or absence of PMA (500 nM). At the indicated time, the cells were centrifuged for 10 min at 600*g*, and metabolism was quenched by addition of ice-cold acetonitrile:water:chloroform solution at a final ratio of 2:1.5:1. After vortexing, the solution was centrifuged at 14,000 rpm for 20 min at 4 °C. Polar fractions were lyophilized using a Freezone 2.5 L −84°C benchtop freeze dryer (Labconco). The dried samples were reconstituted in 200 μl of LCMS grade water with 0.1% formic acid (v/v) (Thermo Scientific; LS118-1), vortexed, and centrifuged at 10,000*g* for 20 min at 4 °C. Then, 75 μl of the supernatant was transferred to a UPLC total recovery glass vial and used for LC–MS analysis.

### LC–MS analysis

All samples were analyzed on an Acquity I-Class UPLC systemoupled to Synapt XS Mass Spectrometer ith MassLynx 4.2 software. The chromatographic separation was achieved using a reversed phase 2.1 mm 150 mm Acquity Premier CSH C18 1.7 μm VanGuard FIT column maintained at 60 °C. The column was eluted with a gradient composed of 0.1% formic acid in water (solvent A) and 0.1% formic acid in acetonitrile (solvent B). The gradient profile started at 0% of B, increased to 7% B over 3.4 min at 0.450 ml/min flow rate, and then increased to 95% B over 6.6 min with a flow rate of 0.600 ml/min. The gradient was held at 95%B for 2.0 min before returning to the initial conditions over 0.05 min, and re-equilibrated for 2.95 min before the next injection.

QTOF–MS data were collected with an electrospray ion source operated in both negative (−) and positive (+) modes. In (−) mode, the capillary voltage was 2.25 kV, the source temperature was 120 °C, the desolvation gas flow was 700 L/h at a temperature of 650 °C, and the cone gas flow was 150 l/h. In (+) mode, the capillary voltage was 0.43 kV, the source temperature was 100 °C, the desolvation gas flow was 1000 l/h at a temperature of 500 °C, and the cone gas flow was 50 l/h. The MS^E^ data-independent acquisition was performed over the *m/z* range of 40−930 Da with low collision energy off (function 1) and high collision energy ramping from 10 to 40 V (function 2) in both ion modes. Each of these functions employed a scan time of 0.2 s. Sodium formate was used for the mass calibration before the sample run. Leucine enkephalin was used as the lock mass solution during the acquisition, which gives the MS signal at *m/z* 554.2615 and 556.2771 in (−) and (+) modes, respectively. After the acquisition, raw files were imported to UNIFI 1.9 software package.

### Stable isotope data analyses

Isotopologue peak deconvolution and assignments were performed using UNIFI 1.9 software package. Peaks were assigned using a metabolite library first generated and verified using full scan MS and tandem MS (MS/MS) spectra of unlabeled samples (49, 50). The library contained metabolite names and corresponding molecular formulae used for generation of theoretical *m/z* values for all possible isotopologues and retention times for each entry. The UNIFI parameters for SIRM library matching were as follows: mass error window ±15 mDa; match retention time window ±0.18 min. For ^13^C isotopologue peak detection, the software criteria were set as follows: maximum number of isotopes per cluster is one, and maximum number of charges for cluster is one in 3D isotope clustering. The assignment table with peak intensities was exported into a Microsoft Excel (.xlsx) file, and all assignments were visually inspected for assignment errors and omissions following guidelines described previously (19). The unlabeled samples were used for reference. Any isotopologue peaks assigned in error, for example, having different retention time than in the unlabeled samples, were deleted. Next, comma-separated (.csv) file was generated with compound name, formula, isotope label, and responses of each sample. Finally, the natural isotopic abundance was corrected using the AccuCore algorithm (51) run using R Script (R version 4.3.1, “Beagle Scouts”). The resulting data were analyzed and plotted with GraphPad Prism 8.0 (GraphPad Software, Inc).

### Statistical analysis

Normality tests were utilized for each dataset to determine the appropriate statistical test. Statistical significance was calculated with Student’s *t* test or ANOVA followed by a Tukey post-test using GraphPad Prism 10. Area under the curve calculations were evaluated utilizing time points after port injection of PMA. For assessment of statistical significance of ^13^C fractional enrichment, data were transformed *via* logit transformation prior to application of statistical tests. A *p* value ≤ 0.05 was considered statistically significant. Data are represented as individual points or mean ± SD.

## Data availability

All ROS data are included in the article. Full metabolomics dataset can be provided upon request. Request data from Tyler Jobe, email: t0jobe02@louisville.edu.

## Supporting information

This article contains supporting information.

## Conflict of interest

The authors declare that they have no conflicts of interest with the contents of this article.

## Supporting information

Supplemental Figs. S1–S4 Caption
