# Supplementary material for: Phase partitioning of the neutrophil oxidative burst is coordinated by accessory pathways of glucose metabolism and mitochondrial activity
Source: J Biol Chem. 2024 Dec 13;301(1):108091. doi: 10.1016/j.jbc.2024.108091 (PMC11760813; doi:10.1016/j.jbc.2024.108091)
Supplement: Supplemental Figs. S1–S4 Caption [file mmc1.docx]

**SUPPLEMENTARY INFORMATION LEGENDS**

**Supplementary Figure 1. Neutrophil purity and viability after sorting.** After magnetic bead sorting neutrophil were stained with eFluor450-Ly6G antibody and PE-Propidium iodide and cell purity and viability was assessed with flow cytometry.

**Supplementary Fig. 2. Multivariate analysis of metabolite profiles in neutrophils. (A-B)** PCA plots displaying the separation of groups after 10 or 60 minutes of incubation with PMA or vehicle (DMSO). **(C-D)** PLSDA plots displaying the separation of groups after 10 or 60 minutes of incubation with PMA or vehicle (DMSO). **(E)** PLSDA plot displaying separation of groups by sex in activated neutrophils after 60 min of PMA stimulation. **(F)** VIP Scores plot displaying the top 10 metabolites that are responsible for group separation in panel E. N = 6 (3 males and 3 females/group), M = male and F = female.

**Supplementary Fig. 3. In glucose-free conditions, glycogen catabolism supports early neutrophil ROS production.** Measurement of the respiratory burst in the absence or presence of glycogen phosphorylase inhibitor: (**A**) Schematic of glycogen contribution to the respiratory burst, showing use of a glycogen phosphorylase inhibitor (GPi) to inhibit glycogen breakdown. (**B**) Oxygen consumption rate (OCR) trace of bone marrow-derived neutrophils incubated in glucose-free media with GPi (100 µM) or vehicle (DMSO) and stimulated with PMA (500 nM). (**C**) Area under the curve analysis of the OCR trace in panel B. N = 6 (3 males and 3 females/group). Statistical significance was assessed via Student’s unpaired T-test.

**Supplementary Figure 4. GAPDH inhibition has no impact neutrophil ROS production even though limits glycolytic flux.** Measurement of the respiratory burst in the absence or presence of glyceraldehyde 3-phosphate dehydrogenase inhibitor: (**A**) Schematic showing site of inhibition of koningic acid (KA). (**B–C**) Oxygen consumption rate (OCR) and proton efflux rate (PER) trace of bone marrow-derived neutrophils incubated in glucose-free media with KA (10 µM) or vehicle (DMSO) and stimulated with PMA (500 nM). (**D–E**) Oxygen consumption rate (OCR) and proton efflux rate (PER) trace of bone marrow-derived neutrophils incubated in glucose-free media with KA (20 µM) or vehicle (DMSO) and stimulated with PMA (500 nM). N = 12 (6 males and 6 females/group). Area under the curve analysis of the OCR trace displayed no significant difference in the treatment group compared to the vehicle.
